# Effects of social housing on alcohol intake in mice depend on the non-social environment

**DOI:** 10.3389/fnbeh.2024.1380031

**Published:** 2024-05-16

**Authors:** Michael C. Johnson, Jonathan A. Zweig, Yangmiao Zhang, Andrey E. Ryabinin

**Affiliations:** Department of Behavioral Neuroscience, School of Medicine, Oregon Health and Science University, Portland, OR, United States

**Keywords:** alcohol drinking, ethanol intake, mice, social housing, social isolation, environmental enrichment

## Abstract

**Background:**

Excessive alcohol consumption leads to serious health problems. Mechanisms regulating the consumption of alcohol are insufficiently understood. Previous preclinical studies suggested that non-social environmental and social environmental complexities can regulate alcohol consumption in opposite directions. However, previous studies did not include all conditions and/or did not include female rodents. Therefore, in this study, we examined the effects of social versus single housing in standard versus non-standard housing conditions in male and female mice.

**Methods:**

Adult C57BL/6 J mice were housed in either standard shoebox cages or in automated Herdsman 2 (HM2) cages and exposed to a two-bottle choice procedure with 3% or 6% ethanol versus water for 5 days. The HM2 cages use radiotracking devices to measure the fluid consumption of individual mice in an undisturbed and automated manner. In both housing conditions, mice were housed either at one or at four per cage.

**Results:**

In standard cages, group housing of animals decreased alcohol consumption and water consumption. In HM2 cages, group housing significantly increased ethanol preference and decreased water intake. There were no significant differences in these effects between male and female animals. These observations were similar for 3 and 6% ethanol solutions but were more pronounced for the latter. The effects of social environment on ethanol preference in HM2 cages were accompanied by an increase in the number of approaches to the ethanol solution and a decrease in the number of approaches to water. The differences in ethanol intake could not be explained by differences in locomotor or exploratory activity as socially housed mice showed fewer non-consummatory visits to the ethanol solutions than single-housed animals. In addition, we observed that significant changes in behaviors measuring the approach to the fluid were not always accompanied by significant changes in fluid consumption, and vice versa, suggesting that it is important to assess both measures of motivation to consume alcohol.

**Conclusion:**

Our results indicate that the direction of the effects of social environment on alcohol intake in mice depends on the non-social housing environment. Understanding mechanisms by which social and non-social housing conditions modulate alcohol intake could suggest approaches to counteract environmental factors enhancing hazardous alcohol consumption.

## Introduction

Alcohol use disorder (AUD) is a prevalent public health problem in the United States and worldwide. It is estimated that alcohol drinking results in 3–6% of annual deaths worldwide and represents a leading cause of preventable death (Sacks et al., 2015; GBD, 2016; Di Castelnuovo et al., 2019; Alcohol-Related Disease Impact, 2023; NIAAA, 2024). Despite significant research efforts and the emergence of several FDA-approved therapeutics, there has been limited success in treating AUD, as hazardous alcohol drinking has continued to rise in recent years (Grant et al., 2017; Daly and Robinson, 2021; Irizar et al., 2021; NIAAA, 2024). Therefore, understanding mechanisms regulating the consumption of alcohol remains an important priority for biomedical research.

Many environmental factors influence alcohol drinking and excessive alcohol consumption. The factors included, and often overlooked, are the influences of social dynamics on alcohol drinking (Heilig et al., 2016; Sudhinaraset et al., 2016; Mac Killop et al., 2022). For instance, during the COVID-19 pandemic, hazardous alcohol drinking rose, likely due to increases in environmental stress and increased social isolation due to lockdowns (Irizar et al., 2021; Killgore et al., 2021; Weerakoon et al., 2021). On the other hand, epidemiological data indicate that social drinking with peers or partners is associated with the facilitation of hazardous alcohol consumption (Weitzman et al., 2003; Kuntsche et al., 2004; Nogueira-Arjona et al., 2019; Monk et al., 2020; Creswell, 2021). A substantial experimental human research literature indicates that social context can facilitate hazardous alcohol consumption through normative (drinking for ingratiation) and informational influences (drinking “because others do”) (Deutsch and Gerard, 1955; Quigley and Collins, 1999; Larsen et al., 2010; Dallas et al., 2014) and that the magnitude of this influence can depend on contextual cues (Tomaszewski et al., 1980; Doty and de Wit, 1995; Dallas et al., 2014; Monk et al., 2018). However, given the difficulty of controlling the alcohol consumption history and individual genetic and developmental differences, there are challenges to infer causality in such studies. Therefore, animal models of social alcohol drinking are critical to understanding the complicated interplay between social dynamics and alcohol consumption.

The majority of studies in rodent models utilize single-housed animals and relatively simple environments, which do not model human conditions. A number of previous studies in rodents have attempted to study the effects of more complex environments, including social housing and enrichment, on addiction and alcohol drinking (Pohorecky, 2010; Logue et al., 2014; Varlinskaya et al., 2015; Camarini et al., 2018; Koskela et al., 2018; Ryabinin and Walcott, 2018; Venniro et al., 2020; Stefaniuk et al., 2023). Various effects have been observed. For example, a comprehensive study comparing the effects of group housing in standard (impoverished) conditions and complex “intellicage” approaches suggested that both social enrichment and non-social environmental enrichment decrease alcohol drinking in male C57 mice (Holgate et al., 2017). However, this study did not include single housing in the complex condition and used lickometer approaches. Although the overall number of licks in lickometer cages correlates with alcohol drinking in many studies (Ford et al., 2005; Griffin et al., 2007; Anacker and Ryabinin, 2013), lick sizes may differ across various fluids, circadian phases, or experimental conditions. To circumvent this potential problem, we have recently adopted the tracking of consummatory behaviors using radio frequency identification (RFID) tags in Heardsman-2 (HM2) cages (Thomsen et al., 2017; Caruso et al., 2021; Fulenwider et al., 2021; Walcott and Ryabinin, 2021). The HM2 cages are typically large, allowing group housing of rodents. Each HM2 cage is equipped with two channels. Each channel leads to a bottle placed on a precision balance. Only one animal can enter the channel at a time. By a combination of an RFID reader and photoelements located in the channels, the consummatory behaviors of each individual rodent housed either singly or in groups can be tracked with high temporal resolution. HM2 cages can be considered more complex housing conditions because consummatory behavior in these cages requires animals to learn to climb into the channels to get access to fluids and make active choices to enter the channels for specific fluids. In contrast, in a standard cage setup, the drinking spouts protrude into the cage and are next to each other, not requiring memorization of the location of the fluid and making the choice of the fluid easy.

Using the HM2 caging system in male C57 mice, we have observed that while single-housed mice display very low alcohol intake in a two-bottle choice (2 BC) procedure, mice housed at four animals per cage had a higher intake of alcohol per mouse (Caruso et al., 2021; Fulenwider et al., 2021). This finding suggested that non-social and social differences in housing conditions can produce varied effects on alcohol intake: non-social complexity of HM2 cages contributes to lower alcohol intake, whereas the social complexity of group housing contributes to increased alcohol intake in these cages. However, this interpretation was hampered by three potential caveats. First, the previous studies did not perform parallel experiments across all four housing conditions: simple environment + single housing, simple environment + group housing, complex environment + single housing, and complex environment + group housing, leaving open the possibility that the mismatch in effects is due to laboratory conditions. Second, the previous studies in mice were performed only in males, and thus did not allow the assessment of the generality of effects. Third, the previous studies did not adjust the cage size between the single and group-housed animals in the large HM2 enclosures, leaving open the possibility that the higher drinking in the group-housed mice was not due to social effects but due to the availability of space per mouse. Therefore, the current study aimed to overcome these caveats.

Specifically, the goals of the study were to definitively determine the effects of social housing on alcohol drinking in male and female mice and to assess the dependence of these effects on the non-social differences between environments. To attain these goals, we first compared 2 BC alcohol drinking in C57 mice housed either at one or at four per cage in the standard shoebox cages. Having observed social inhibition, rather than facilitation, of alcohol drinking in these relatively “simple” housing conditions, we then compared 2 BC alcohol drinking in C57 mice housed either at one or at four per cage in HM2 cages. We adjusted the space available to each animal in HM2 cages between the single and group-housed conditions to evaluate whether the observed effects were due to social factors. Taking advantage of the full capabilities of the HM2 system, we also analyzed various behaviors that contributed to differences in alcohol drinking across housing conditions.

Experiments were performed in male and female mice, and the results demonstrated a lack of sex differences in facilitating the effects of social housing on alcohol drinking in HM2 cages.

## Methods

### Animals

All experiments were approved by the Institutional Animal Care and Use Committee at OHSU, Portland, OR, United States and were conducted in accordance with the National Institutes of Health (NIH) Guidelines for the Care and Use of Laboratory Animals. Adult C57BL/6 J mice (total 87 males and 78 females) were purchased from the Jackson Laboratory (Sacramento, CA) with mice at 8 weeks of age. Upon arrival, mice were housed in sex-matched groups of four in standard shoebox cages (18.4 × 29.2 × 12.7 cm) with food and water available *ad libitum*. All cages contained one cotton nestlet and paper nesting materials. After 48 h, male and female mice were transferred to experimental housing and social conditions for 2 BC drinking experiments. Experimental rooms were kept on a 12:12 light/dark cycle (lights off at 11:00).

### Standard shoebox housing

**Figure 1:**
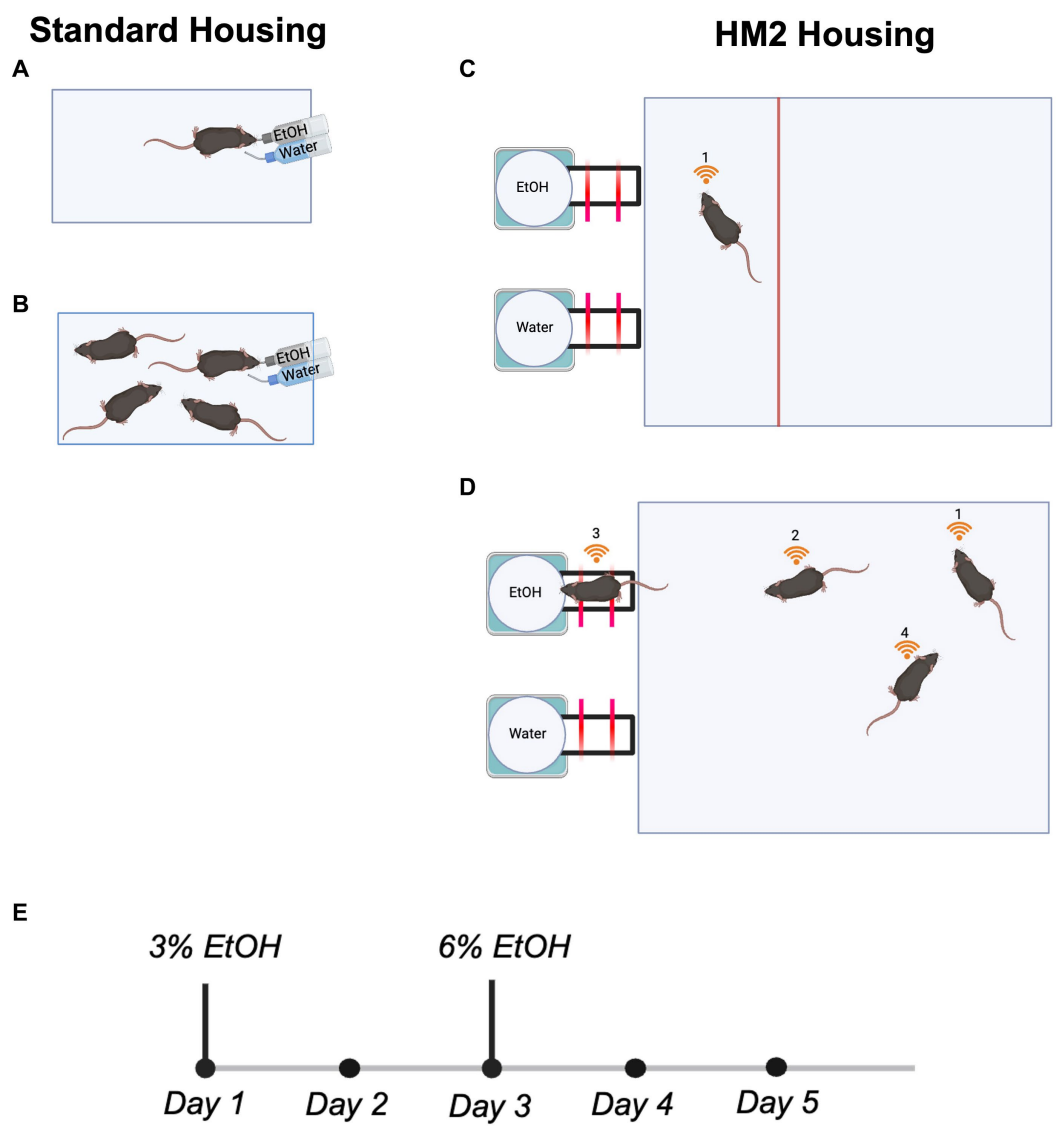
Housing conditions and the experimental timeline for all 2 BC experiments. **(A)** Single mouse housed in a standard shoebox cage. **(B)** Four mice housed in a standard shoebox cage. **(C)** Single mouse housed in a truncated HM2 cage. **(D)** Four mice housed in an HM2 cage. **(E)** Experimental timeline.

In standard housing conditions, mice were housed in standard shoebox cages (18.4 × 29.2 × 12.7 cm). All cages contained one cotton nestlet and paper nesting materials. Per social conditions, mice were either housed individually (*n* = 8 males and 12 females; Figure 1A) or in sex-matched groups of four (*n* = 16 males and 8 females; Figure 1B).

### Herdsman2 (HM2) housing

Mice were housed in larger HM2 cages (48 × 37.5 × 21 cm; described in Caruso et al., 2021). The moderate environmental complexity provided by these housing units relative to standard shoebox cages consists of a larger traversable surface area for group-housed animals and two built-in channels that lead to bottled fluid (ethanol or water) at the end. All cages contained one cotton nestlet and paper nesting materials. Per social conditions, mice were either housed in isolation (*n* = 11 males and 11 females; Figure 1C) or in sex-matched groups of four (*n* = 32 males and 36 females; Figure 1D). Single-housed mice had a partition installed into the HM2 cage to control the disproportionate size of the HM2 cage relative to a single mouse (48 × 12 × 21 cm; Figure 1C).

### RFID implantation

Directly preceding transfer to 2 BC experimental housing, all mice housed in HM2 cages (*n* = 43 males and 47 females) were lightly anesthetized using isoflurane, and RFID chips (UNO MICRO ID/7, ISO Transponder 2.12 × 7 mm, Med Associates, Fairfax, VT, United States) were inserted subcutaneously behind their shoulder blades. Successful RFID insertion was validated using an HM2 RFID scanner (MBRose, Faaborg, Denmark).

### Two-bottle choice paradigm

Mice in both housing conditions underwent 5 days of water habituation following transfer to experimental housing conditions, during which water was continuously available from both bottles in their cages. Following this, all mice were transitioned to a 2 BC paradigm, where one bottle in their cage contained 3% ethanol in autoclaved water, and the other contained only autoclaved water. This continued for 2 days, after which, 3% ethanol bottles were swapped to 6% ethanol in autoclaved water for the next 3 days (Figure 1E). We did not use higher concentrations of ethanol because our previous experiments indicated that mice strongly reduced intake of ethanol in HM2 cages when higher than 6% solutions were used (Caruso et al., 2021; Fulenwider et al., 2021). In standard shoebox cages, the position of ethanol and water bottles were swapped daily to avoid side preference bias. In HM2 cages, bottle sides were varied for every cage in every experiment to avoid side preference bias.

The data on fluid consumption in HM2 cages is registered as the weight of fluid consumed. The HM2 system is built such that it measures only fluid consumed by the animal. Evaporation is taken into account because weight measures are taken at the time of consumption. The spillage of fluid is taken into account by a catch tray that is also weighed but not included in the consumption data. This feature presents a substantial advantage over home cage drinking measurements and lickometer systems, which cannot take the spilled fluid into account. 2 BC data were analyzed as grams of ethanol and grams of water per kilogram bodyweight consumed. In addition to allowing high temporal resolution RFID tracking of fluid intake, HM2 cages also provide detailed information regarding visits to fluid channels for each mouse. Among these are the two measures of approach to the corresponding fluids: consummatory visits (CV), the number of visits to a fluid channel that resulted in fluid consumption, and consummatory visit time (CV time), the time spent in the channel during a particular time interval. It also includes two measures reflecting exploratory or locomotor activity: non-nutritious visits (NNV), the number of visits to a fluid channel that did not result in fluid consumption, and non-nutritious visit time (NNV time), the time spent in the channel during a particular time interval. All these behaviors were measured in HM2 cages for each social condition during 2 BC. Preference measures were also calculated as intake (in ml) or approaches specific for each fluid in HM2 cages (ethanol intake, ethanol CV, ethanol CV time, ethanol NNV, ethanol NNV time divided by total fluid intake, total fluid CV, total fluid CV time, total fluid NNV, and total fluid NNV time). After the completion of the experiment, to determine if cage effects could have contributed to observed differences in ethanol intake or preference in HM2 cages, all animals were divided into equally sized high-, medium-, and low-alcohol drinking subgroups or high-, medium-, and low-alcohol preferring subgroups based on average measures at the 6% ethanol concentration. While individualized consumption for grouped mice in HM2 cages could be tracked as animals that were RFID tagged, it could not be tracked in standard housing. Thus, for four mice housed in standard shoebox cages, total consumption within each standard cage was divided by four mice and by the weight of each animal within the cage. Preference measures were not calculated for the shoebox housing conditions as they would result in no variability in group-housed mice.

### Statistical analyses

All data are presented as means ± standard error of the mean. SPSS and GraphPad Prism were used for data analysis and to generate graphs, respectively. All measures were averaged across either 2 days of 3% or 3 days of 6% ethanol. Two male mice and one female mouse were removed from the study due to RFID failures. Grubb’s outlier test was performed on the raw data of every group for all 3 and 6% average daily measures prior to analysis. No outliers were removed from hourly repeated measures and no more than 1 outlier per group was removed from 1 to 2 (3%) day and 3–5-day (6%) average measures. Two-way ANOVAs (social condition by sex or sex by cage) were performed on collected measures at each ethanol concentration.

To evaluate whether there was a shift in the circadian cycle associated with differences in fluid intake in the group-housed versus single-housed mice, average ethanol and water intake was also graphed by hourly consumption over 24 h and analyzed by repeated ANOVA (between-subject factors: sex and social condition, within-subject factor: time). If warranted, by a significant main effect or interaction, Tukey’s *post-hoc* tests were conducted. Bonferroni’s tests were used for pairwise comparisons of 24 h data. For the clarity of reading, the results section only presents *p* values of significant tests. All other statistical outcomes are reported in the Supplementary Statistics Table.

## Results

### Daily average ethanol and water intake during 2 BC in standard cages

**Figure 2:**
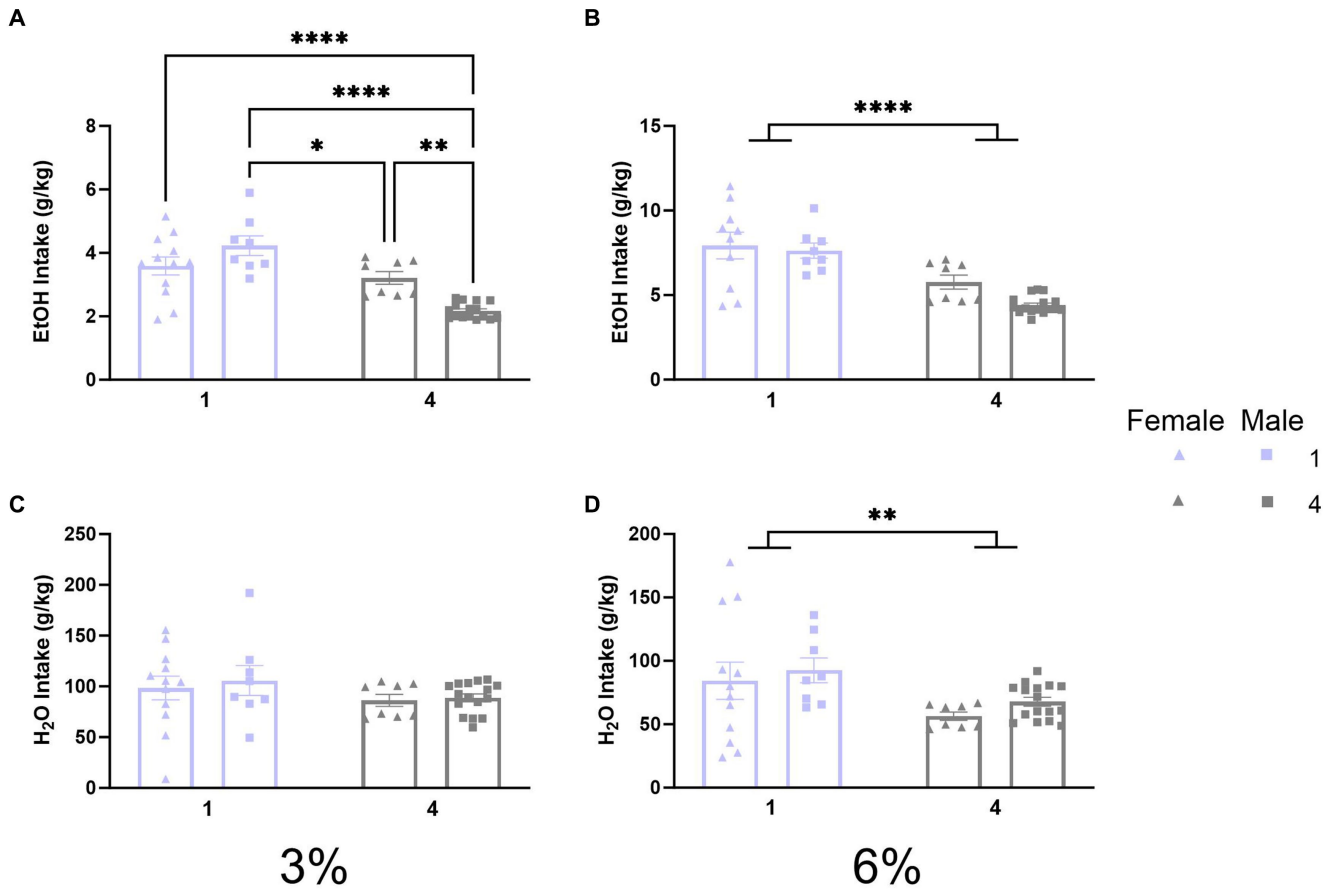
EtOH and H_2_O intake across 2 BC in shoebox cages with varied social conditions. **(A)** Ethanol intake (g/kg) for days 1–2 (3%). *p < 0.05; **p < 0.01; ***p < 0.001; ****p < 0.0001 between groups in Tukey’s *post-hoc* test. **(B)** Ethanol intake (g/kg) for days 3–5 (6%). ****Indicates significant main effect of social condition (*p* < 0.0001). **(C)** Water intake (g/kg) for days 1–2 (3%). **(D)** Water intake (g/kg) for days 3–5 (6%). **Indicates significant main effect of social condition (*p* < 0.01).

Previously, 2 BC experiments in HM2 cages revealed that social housing increases ethanol drinking compared to single-housed counterparts in male mice in HM2 cages (Fulenwider et al., 2021). The initial experiment of the present study tested whether similar effects can be observed in standard shoebox cages. Therefore, we compared ethanol and water intake (g/kg) between single-housed and four-housed male and female mice. A two-way ANOVA with factors of sex and social condition on 3% ethanol intake revealed a significant interaction of social condition by sex (*p* < 0.0005, Figure 2A). Tukey’s *post-hoc* tests revealed that socially housed males had the lowest ethanol intake compared to all other groups (*p* < 0.001) whereas single-housed females had higher ethanol intake than socially housed males (*p* < 0.05). Meanwhile, a two-way ANOVA on 6% ethanol intake revealed a significant main effect of social condition (*p* < 0.0001, Figure 2B), indicating that single-housed mice consumed more ethanol than socially housed mice. Analysis of water intake during the 3% period with a two-way ANOVA detected no significant differences between groups (Figure 2C). However, a two-way ANOVA of water intake during the 6% period revealed a significant effect of social condition (*p* < 0.01, Figure 2D) indicating that single-housed mice also consumed more water than socially housed mice during the 6% ethanol period.

Since intake values in the social housing condition were estimated by dividing total cage fluid intake by the number of mice in each cage and their specific body weights, it would be impossible to statistically compare preferences for individual mice houses in the social condition. Therefore, the preference was not calculated. Overall, this first experiment revealed that social housing in standard cages decreases alcohol intake and does not replicate the facilitating effects of social housing on alcohol intake observed previously in HM2 cages. Thus, the facilitatory effects of social housing on alcohol intake appear to depend on the non-social housing environment.

### Daily average ethanol and water intake during 2 BC in HM2 cages

**Figure 3:**
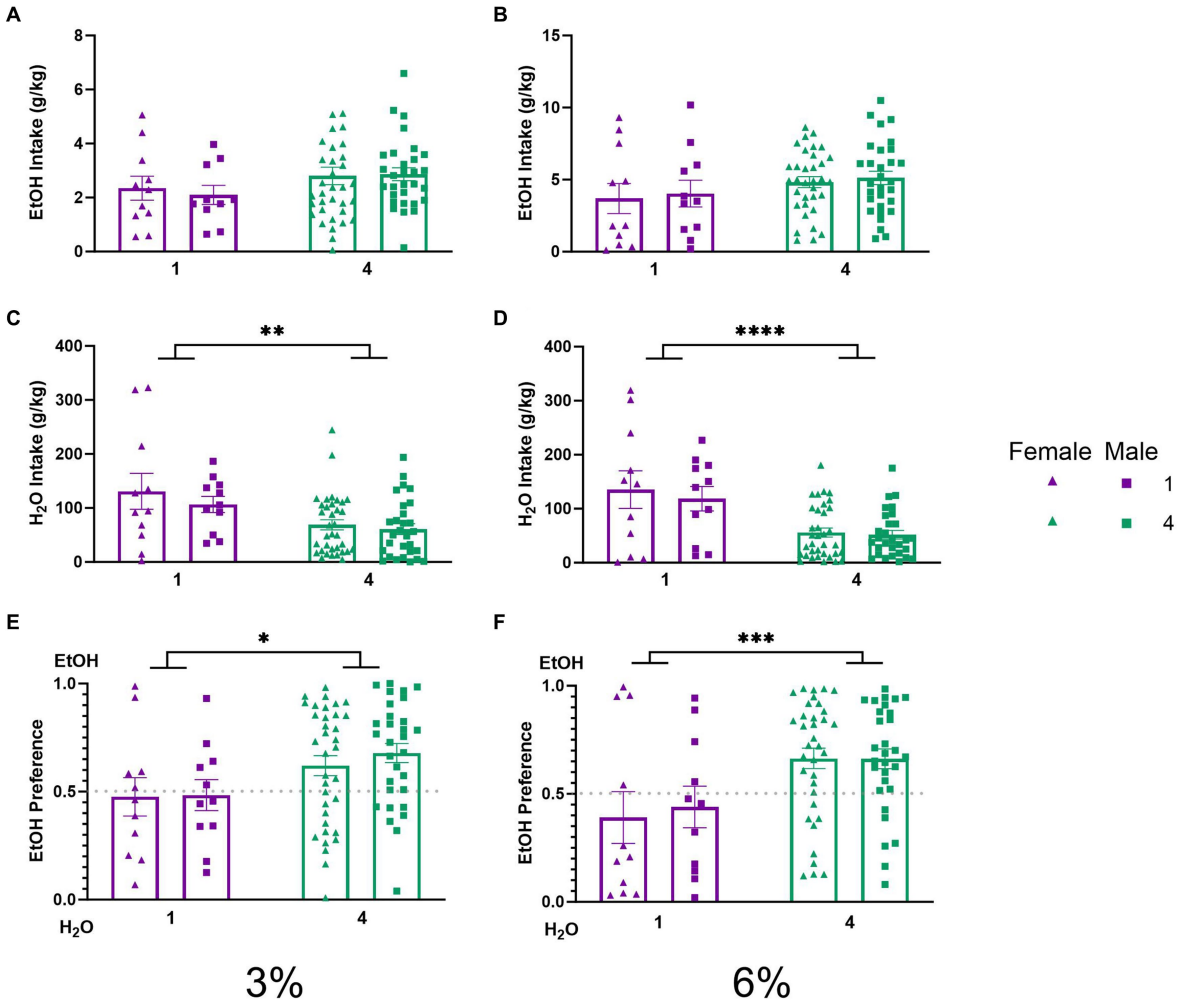
EtOH and H_2_O intake across 5 days of 2 BC in HM2 cages with varied social conditions. **(A)** Ethanol intake (g/kg) for days 1–2 (3%). **(B)** Ethanol intake (g/kg) for days 3–5 (6%). **(C)** Water intake (g/kg) for days 1–2 (3%). **(D)** Water intake (g/kg) for days 3–5 (6%). ****Indicates significant main effect of social condition (*p* < 0.0001). **(E)** Ethanol preference for days 1–2 (3%). *Indicates significant main effect of social condition (*p* < 0.05). **(F)** Ethanol preference for days 3–5 (6%). ***Indicates significant main effect of social condition (*p* < 0.001).

To characterize the effects of social housing on voluntary ethanol drinking in the HM2 cages, average ethanol intake (g/kg) and water intake (g/kg) were compared between single and group-housed male and female mice. Two-way ANOVAs with factors of social condition and sex, on average intake of 3 and 6% ethanol, respectively, did not detect significant differences between groups. (Figures 3A,B). Meanwhile, two-way ANOVAs with factors of social condition and sex, on average water intake during 3 and 6% alcohol drinking period revealed significant main effects of social condition (*p* < 0.01, Figure 3C; *p* < 0.0001, Figure 3D). To further characterize these observations, the average ethanol preferences during 3 and 6% ethanol periods were compared. A two-way ANOVA revealed a significant main effect of social condition for both 3 and 6% drinking periods (*p* < 0.05, Figure 3E; *p* < 0.001, Figure 3F), indicating that socially housed mice in HM2 cages had a significantly higher preference for ethanol over water than their singly housed counterparts.

### Average daily consummatory visits during 2 BC in HM2 cages

**Figure 4:**
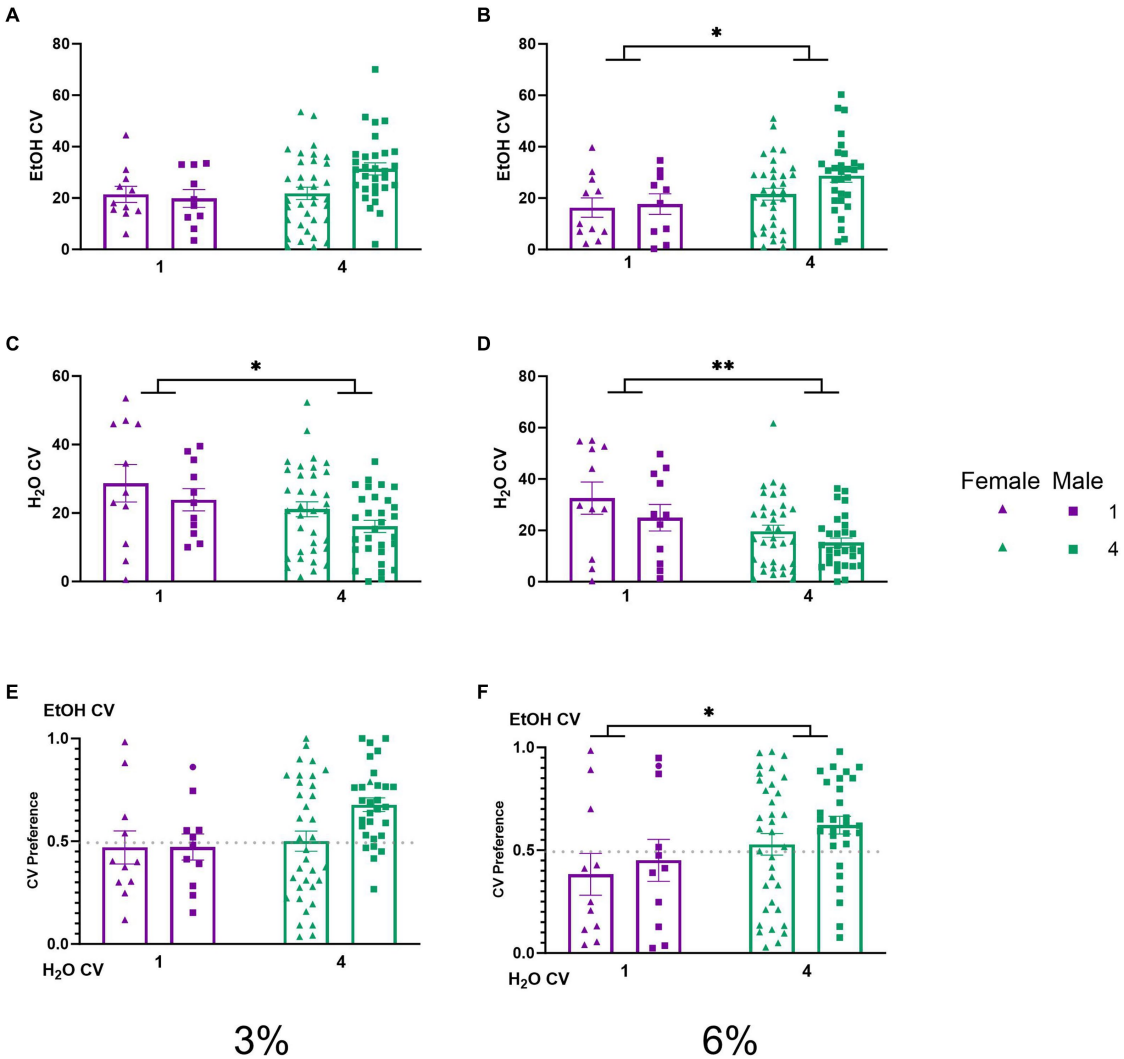
EtOH and H_2_O CVs across 5 days of 2 BC in HM2 cages with varied social conditions. **(A)** Ethanol CVs for days 1–2 (3%). **(B)** Ethanol CVs for days 3–5 (6%). *Indicates significant main effect of social condition (*p* < 0.05). **(C)** Water CVs for days 1–2 (3%). *Indicates significant main effect of social condition (*p* < 0.05). **(D)** Water CVs for days 3–5 (6%). **Indicates a significant main effect of social condition (*p* < 0.01). **(E)** Ethanol CV preference for days 1–2 (3%). **(F)** Ethanol CV preference for days 3–5 (6%). *Indicates significant main effect of social condition (*p* < 0.05).

To understand the behavioral mechanisms that lead to the social facilitation of ethanol preference observed above, we analyzed the CV and CV time of mice under different social conditions. No significant effects on ethanol CVs were detected during the 3% drinking period (Figure 4A). A two-way ANOVA of average number of ethanol CVs during the 6% drinking period revealed a significant effect of social condition (*p* < 0.05, Figure 4B), indicating that socially housed mice had higher ethanol CVs than singly housed mice. Two-way ANOVAs of water CVs during the 3 and 6% periods each revealed significant main effects of social conditions, respectively (*p* < 0.05, Figure 4C; *p* < 0.005, Figure 4D), where socially housed mice had lower CVs to the water channel than single-housed mice during both drinking periods. We next compared the average ethanol CV preference for each group across both the 3 and 6% drinking periods. A two-way ANOVA of ethanol CV preference during the 3% drinking period produced a trend for the effect of social condition that did not reach statistical significance (*p* = 0.055, Figure 4E). A two-way ANOVA of ethanol CV preference during the 6% drinking period revealed a significant main effect of social condition (*p* < 0.05, Figure 4F), indicating that socially housed mice had a higher preference for ethanol CVs over water CVs than singly housed mice.

**Figure 5:**
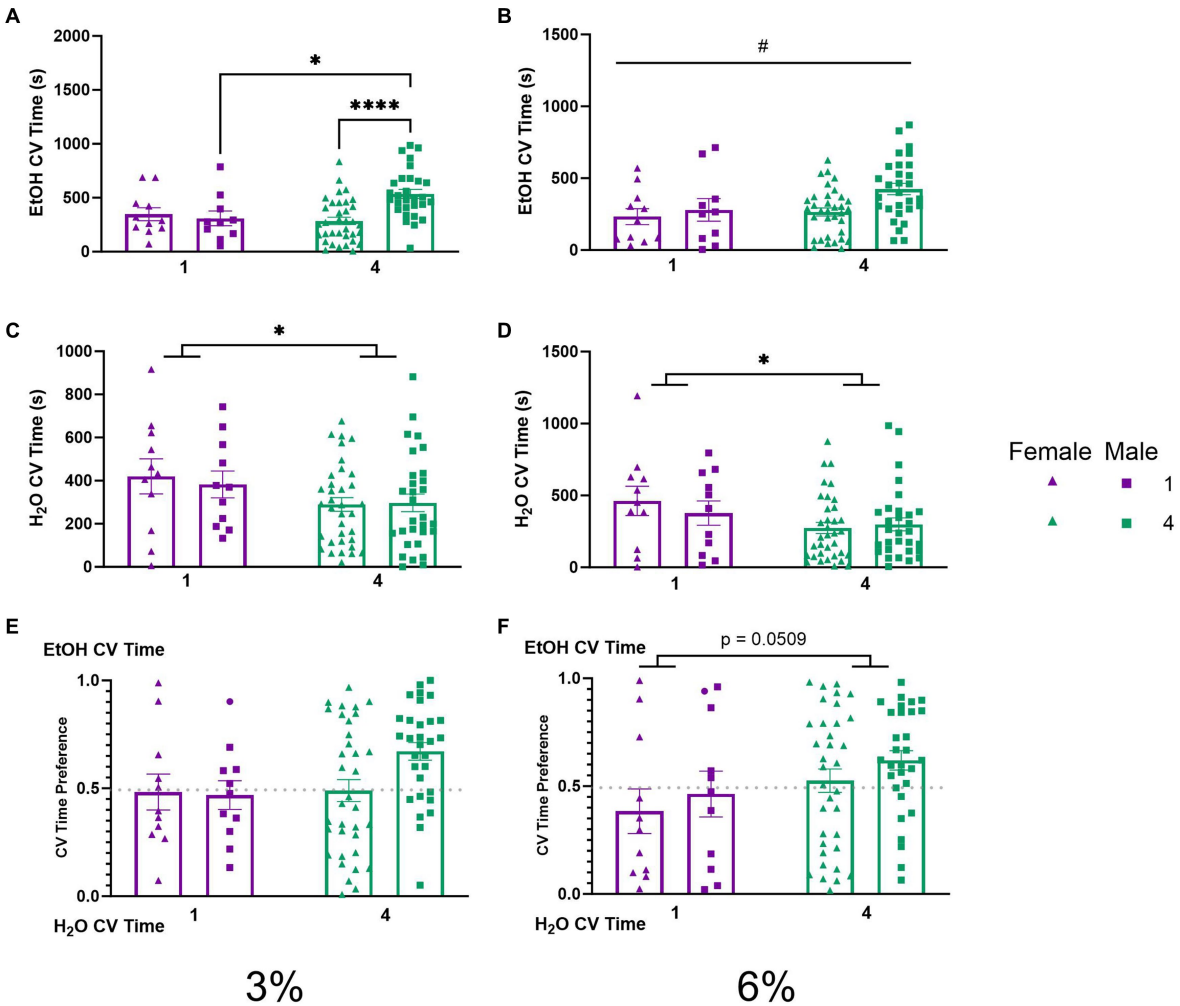
EtOH and H_2_O CV time across 5 days of 2 BC in HM2 cages with varied social conditions. **(A)** Ethanol CV time for days 1–2 (3%) (%). **, ****Indicates a significant difference between groups in Tukey’s *post-hoc* test (*p* < 0.01, 0.0001). **(B)** Ethanol CV time for days 3–5 (6%). #Indicates significant main effect of sex (*p* < 0.05). **(C)** Water CV time for days 1–2 (3%). *Indicates significant main effect of social condition (*p* < 0.05). **(D)** Water CV time for days 3–5 (6%). *Indicates significant main effect of social condition (*p* < 0.05). **(E)** Ethanol CV time preference for days 1–2 (3%). **(F)** Ethanol CV time preference for days 3–5 (6%). The effect of social conditions failed to reach significance (*p* = 0.0509).

To further probe whether differences in ethanol preference (Figures 3E,F) could be attributable to the amount of time spent during drinking these visits, we compared CV time (total time spent during CVs) for ethanol and water channels across our groups. A two-way ANOVA of ethanol CV time during the 3% drinking period revealed a significant interaction of social condition by sex (*p* < 0.01, Figure 5A). Tukey’s *post-hoc* tests confirmed that socially housed males had significantly higher ethanol CV time than singly housed males (*p* < 0.05) and socially housed females (*p* < 0.0001), respectively. During the 6% drinking period, a two-way ANOVA of ethanol CV time revealed a significant effect of sex (*p* < 0.05, Figure 5B) with males having higher ethanol CV time than females. Meanwhile, two-way ANOVAs of water CV time during the 3 and 6% drinking periods both revealed significant main effects of social condition, respectively (*p* < 0.05, Figure 5C; *p* < 0.05, Figure 5D), indicating that socially housed mice maintained lower water CV time compared to singly housed mice during both drinking periods. The two-way ANOVA on ethanol CV time preference detected no significant effects (Figures 5E,F). However, there was a trend toward an effect of social condition (*p* = 0.0509, Figure 5F) during the 6% drinking period, in line with the above-described effects on CV preference (Figure 4F).

### Non-nutritious visits during 2 BC in HM2 cages

**Figure 6:**
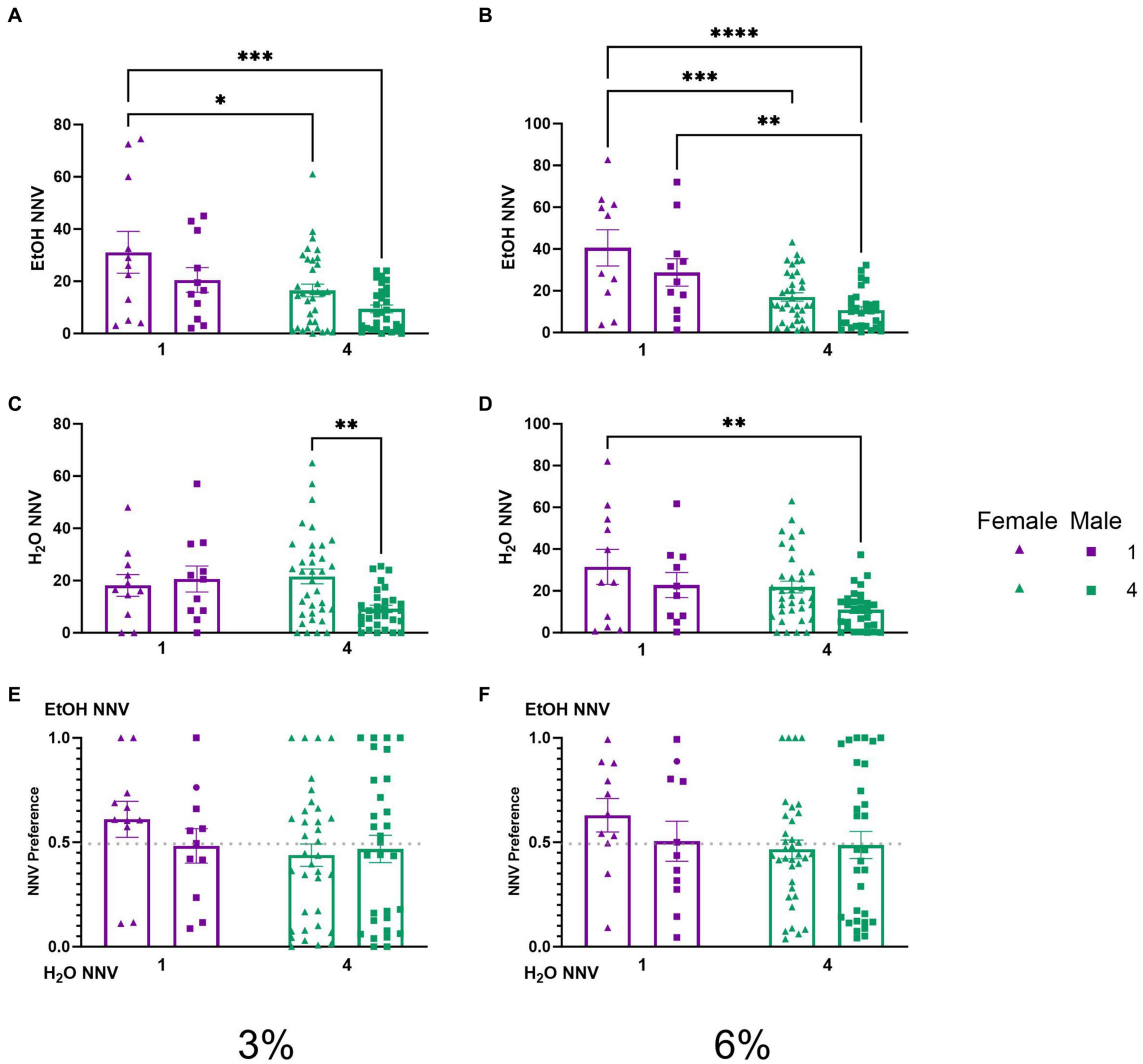
EtOH and H_2_O NNVs across 5 days of 2 BC in HM2 cages with varied social conditions. **(A)** Ethanol NNVs for days 1–2 (3%). *, ***Indicates a significant difference between groups in Tukey’s *post-hoc* test (*p* < 0.05, 0.001). **(B)** Ethanol NNVs for days 3–5 (6%). **, ***, ****Indicates a significant difference between groups in Tukey’s *post-hoc* test (*p* < 0.01, 0.001, 0.0001). **(C)** Water NNVs for days 1–2 (3%). **Indicates a significant difference between groups in Tukey’s *post-hoc* test (*p* < 0.01). **(D)** Water NNVs for days 3–5 (6%). **(E)** Ethanol NNV preference for days 1–2 (3%). **(F)** Ethanol NNV preference for days 3–5 (6%).

To test whether the effects of social housing on consummatory visits observed above were a reflection of higher locomotor or exploratory activity of socially housed mice, we analyzed non-nutritious visits. A two-way ANOVA of ethanol NNV time during the 3% drinking period revealed significant effects of sex (*p* < 0.01) and social condition (*p* < 0.001, Figure 6A). Tukey’s *post-hoc* tests revealed that single-housed female mice had higher ethanol NNVs than both socially housed females (*p* < 0.05) and socially housed males (*p* < 0.001), respectively. A two-way ANOVA of average ethanol NNVs during the 6% drinking period revealed significant main effects of sex (*p* < 0.05) and social condition (*p* < 0.0001, Figure 6B). Tukey’s *post-hoc* tests revealed that female single-housed mice had significantly higher ethanol NNVs than both socially housed females (*p* < 0.001) and socially housed males (*p* < 0.0001), respectively, whereas male single-housed mice had significantly higher ethanol NNVs than socially housed males (*p* < 0.01). Meanwhile, a two-way ANOVA of water NNVs during the 3% period revealed a significant interaction of social condition by sex (*p* < 0.05, Figure 6C). Tukey’s *post-hoc* tests revealed that socially housed females had higher water NNVs than socially housed males during the 3% drinking period (*p* < 0.01). A two-way ANOVA of water NNVs during the 6% drinking period revealed significant main effects of social condition (*p* < 0.05) and sex (*p* < 0.05) (Figure 6D). Tukey’s *post-*hoc tests indicated that single-housed females had significantly higher water NNVs than socially housed males (*p* < 0.01). Reflecting decreases in both ethanol and water NNV, we did not observe differences in preferential NNVs (Figures 6E,F). These data indicate that the previously described difference in consummatory visits between socially and single-housed mice is not due to the overall greater activity in these animals. Instead, both males and females decreased the number of non-consummatory visits to the channels when socially housed.

**Figure 7:**
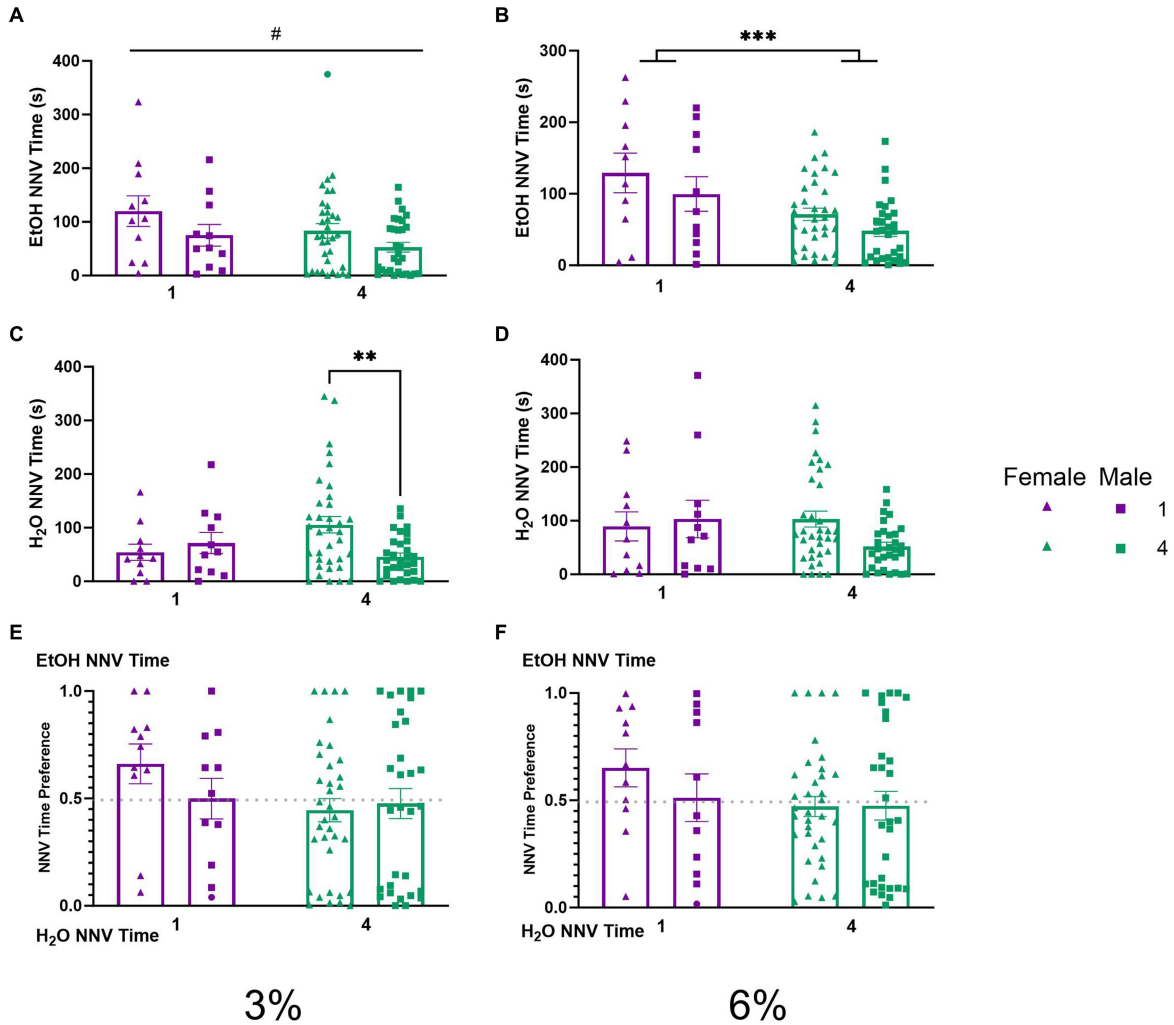
EtOH and H_2_O NNV time across 5 days of 2 BC in HM2 cages with varied social conditions. **(A)** Ethanol NNV time for days 1–2 (3%). #Indicates significant main effect of sex (*p* < 0.05). **(B)** Ethanol NNV time for days 3–5 (6%). ***Indicates significant main effect of social condition (*p* < 0.001). **(C)** Water NNV time for days 1–2 (3%). **Indicates a significant difference between groups in Tukey’s *post-hoc* test (*p* < 0.01). **(D)** Water NNV time for days 3–5 (6%). **(E)** Ethanol NNV time preference for days 1–2 (3%). **(F)** Ethanol NNV time preference for days 3–5 (6%).

To explore the temporal aspects of previously described NNVs between groups, we additionally analyzed NNV time. A two-way ANOVA of 3% ethanol NNV time revealed a significant effect of (*p* < 0.05, Figure 7A) females having higher ethanol NNV time than males. During the 6% drinking period, a two-way ANOVA of ethanol NNV time revealed a significant main effect of social condition (*p* < 0.0005, Figure 7B), indicating that single-housed mice had significantly higher ethanol NNV time than their socially housed counterparts. Meanwhile, a two-way ANOVA of water NNV time during the 3% drinking period revealed a significant interaction of social conditions by sex. Tukey’s *post-hoc* tests indicated that socially housed female mice had significantly higher water NNV time than socially housed male mice (*p* < 0.01, Figure 7C). Analysis of water NNV time during the 6% drinking period did not indicate any significant differences between groups (Figure 7D). Finally, we analyzed the ethanol NNV preferences for the 3 and 6% drinking periods and found no significant effects across social condition or sex (Figures 7E,F).

### Hourly fluid intake

It could be theorized that increased ethanol preference displayed by socially housed mice could be explained by changes in the circadian timing of ethanol or water intake. To test this possibility, we also tracked fluid consumption during the 3 and 6% ethanol periods at an hourly resolution across 24 h. The lights off time was designated as zero hour for this analysis.

**Figure 8:**
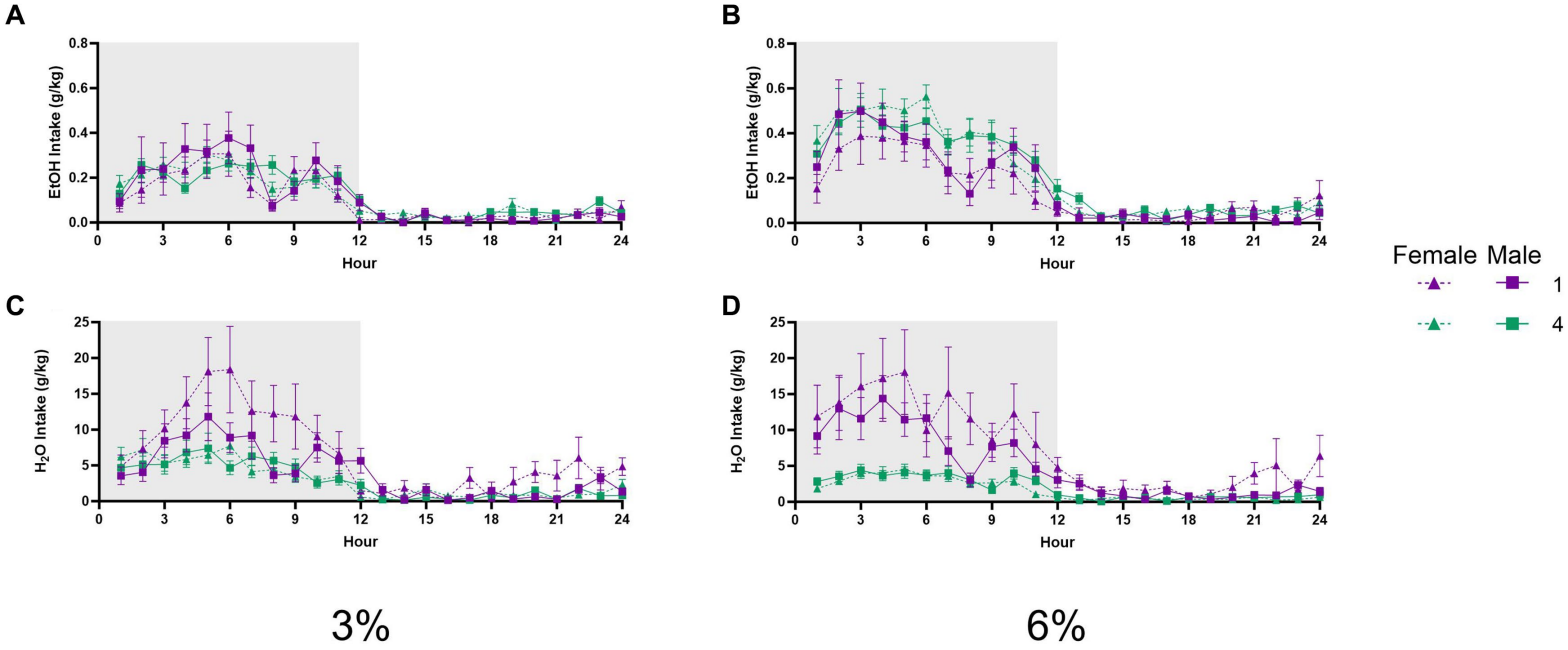
EtOH and H_2_O hourly intake during 2 BC in HM2 cages with varied social conditions. **(A)** Hourly ethanol intake (g/kg) for days 1–2 (3%). **(B)** Hourly ethanol intake (g/kg) for days 3–5 (6%). **(C)** Hourly water intake (g/kg) for days 1–2 (3%). **(D)** Hourly water intake (g/kg) for days 3–5 (6%).

A three-way repeated measures ANOVA with factors of social condition and sex on average hourly ethanol intake during 3% ethanol days across 24 h revealed an interaction of hour by social condition (*p* < 0.01, Figure 8A). A three-way repeated measures ANOVA of average hourly ethanol intake during the 6% drinking period revealed a significant effect of the hour (*p* < 0.0001, Figure 8B); however, no interactions with other effects were detected. Meanwhile, a three-way repeated measures ANOVA on average hourly water intake during the 3 and 6% drinking periods both revealed significant interactions of the hour by sex by social condition (*p* < 0.005, Figure 8C; *p* < 0.01 Figure 8D). These significant interactions suggested that differences in water intake between groups and sexes could vary across time points of the 24 h cycle. In particular, we noted that at the 8 h time point of the 6% drinking period, there was a different relationship in water intake between groups versus other points of the circadian cycle. *Post-hoc* analysis of water intake at this time point revealed that singly housed female mice had higher water intake than four-housed males (*p* < 0.01). Taken together, these data did not indicate that social housing or sex resulted in shifts in the circadian timing of ethanol or water drinking that could have contributed to differences in alcohol preference or intake detected by the analyses of 24 h data.

### Distribution of social drinking data across cages

**Figure 9:**
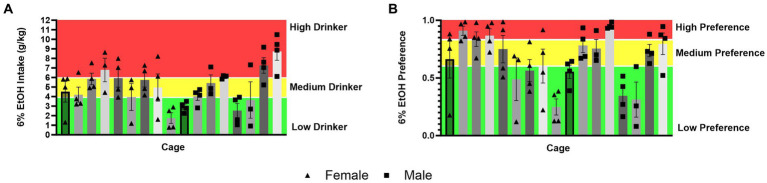
Distribution of ethanol intake (g/kg) and ethanol preference by a cage in four-housed mice in HM2 cages. **(A)** Ethanol intake (g/kg) across cages of four-housed mice. **(B)** Ethanol preference across cages of four-housed mice.

It could also be theorized that differences in ethanol intake and alcohol preference in mice socially versus single-housed mice in HM2 cages are due to mice matching each other’s behavior within each cage, possibly resulting in cage effects. To visually assess this possibility, we used the data for the 6% ethanol period of the experiment to divide all socially housed mice animals into high-, medium- and low-alcohol drinking subgroups, or high-, medium-, and low-alcohol preferring subgroups of equal size. While there was a statistically significant effect of the cage on ethanol intake (*p* = 0.0001), there were only two out of 17 cages where mice belonged to only the low-drinking subgroup, while all other cages contained animals of at least two subgroups (Figure 9A). Similarly, when there was a significant effect of the cage on alcohol preference (*p* < 0.0001), there was only one cage where all animals belonged to the high alcohol-preferring subgroup and two cages where animals belonged only to the low alcohol subgroup, while the remaining 14 cages contained animals of at least two subgroups (Figure 9B). Therefore, differences in alcohol preference between cages could not explain increased preference in socially housed versus single-housed mice.

## Discussion

The experiments herein provide evidence that the effects of social factors on voluntary ethanol consumption in mice depend on the non-social properties of the housing environment. Specifically, in standard cages, single-housed mice consume more alcohol than group-housed mice (Figure 2A). In contrast, in HM2 cages, group-housed mice show higher alcohol preference (Figures 3E,F) and make more approaches to consume from the ethanol-containing bottles than single-housed mice (Figure 4B). This facilitating effect of group housing on alcohol preference and approach to ethanol was independent of the sex of the animals.

The vast majority of studies investigating voluntary alcohol consumption in rodents use single-housed animals in standard shoebox cages. While these housing conditions allow easy assessment of voluntary alcohol intake, there are substantial caveats to the interpretation of these studies. Most importantly, single housing is well known to be stressful to rodents, resulting in increased reactivity of the hypothalamic–pituitary–adrenal axis and increased signs of anxiety and depression (Berry et al., 2012; Krügel et al., 2014; Ieraci et al., 2016). Therefore, despite this standard practice, alcohol consumption in single-housed mice should hardly be taken as a “baseline” condition. The lower alcohol consumption in group-housed mice in these cages observed here is in agreement with a substantial number of previous reports (Thiessen and Rodgers, 1965; Deatherage, 1972; Parker and Radow, 1974; Advani et al., 2007; Lopez et al., 2011; Lopez and Laber, 2015; Panksepp et al., 2017). This decrease in alcohol intake can be theorized to reflect lower stress levels in socially housed animals. However, the relationship between stress and alcohol intake in rodents is complex, and exposure to stress can also decrease, instead of increase, alcohol intake (van Erp et al., 2001; Norman et al., 2015). An alternative possibility is that the presence of cagemates within the tight space of standard cages creates competition for the drinking cylinders. In agreement with this idea, the inhibitory effects of group housing on intake of 3% ethanol in our study were more pronounced in male than in female mice (Figure 2A). This sex difference could reflect typically higher levels of aggression in male versus female rodents (Hashikawa et al., 2018; Karamihalev et al., 2020; Fulenwider et al., 2022), which could result in increased competition for resources. Also in agreement with this interpretation, social housing decreased consumption of not only 6% ethanol but also water in the second phase of the experiment in standard housing conditions (Figures 2B,D).

Another caveat of interpreting results in shoebox cages is the possibility that animals are spilling the fluid, in addition to consuming it. This possibility decreases the precision of measurements. We cannot exclude the possibility that spillage is greater in group-housed animals versus single-housed mice. Related to this caveat, measurements of alcohol intake by group-housed mice in standard shoebox cages are also not precise because the intake values were estimated based on the total fluid consumed by the cage and the body weight of each of the four mice in each cage (see Methods). However, since we detected a statistically significant decrease in ethanol intake in the social housing condition in standard shoebox cages compared to single-housed animals (Figure 2B), it is unlikely that the overall decrease in ethanol consumed per cage would have masked social facilitation of this intake in a proportion of mice. The limitations of studying voluntary social alcohol consumption in standard shoebox cages and the opposite effects of social housing on ethanol intake in standard versus HM2 cages demonstrate the utility of the HM2 cages for studying social facilitation of drinking.

The social facilitation of alcohol preference was visible compared to low alcohol preference (Figures 3E,F) and intake (Figures 3C, D) in mice single housed in HM2 cages. We have previously also observed low alcohol intake in single-housed male mice in these cages (Fulenwider et al., 2021). The intakes of alcohol in HM2 cages in the current study were similar to the ones observed in the previous study (4 g/kg/day on average at the 6% phase of the experiment Figure 3B) and substantially lower than the intake of approximately 7.5 g/kg/day observed in standard shoebox cages (Figure 2B). Therefore, prior to discussing why social housing enhances alcohol preference in these cages, we need to consider why alcohol intake is low in these cages compared to the standard shoebox conditions in single-housed mice. The lower intake of alcohol in HM2 cages versus standard cages cannot be attributed to the differences in the sizes of the cages because the intake of alcohol in the HM2 system was the same whether we used the full-size HM2 enclosures for single-housed mice [as in Fulenwider et al. (2021)] or decreased their size using a partition (current experiment). However, importantly, fluid consumption in shoebox cages and HM2 cages could be guided by very different behavioral mechanisms. In the shoeboxes, the spouts of the two drinking cylinders protrude into the center of the cage and are located close to each other (8.5 cm distance). Since the position of the ethanol-containing cylinder is alternated daily, there is no need for the mouse to remember the position of the preferred fluid. As a result, the choice of intake in this type of housing is most likely based on daily sampling. In contrast, the entrances to the channels in HM2 cages are located much further apart (27 cm), and the mouse must climb into and through the channel to reach the drinking spout. Since the fluid at the end of the channel is not alternated from day to day, the mouse is more likely to rely on its memory to access the fluid of choice. This behavior in HM2 cages, therefore, is more likely to demand a more deliberate action from the animal without being able to rely on simple sampling of fluids. As a result, mice could be less efficient at reaching their fluid of choice than in the shoebox cages. The HM2 cages could be, therefore, considered a more complex environment than shoebox cages not because of any special complexity of the cage, but because consummatory behaviors in these cages require more effort.

Interestingly, although intakes of ethanol were similar between the current and previous experiment using different sizes of HM2 enclosures, water intake, and as a result, preference for ethanol was different. In the previous experiment, using unpartitioned HM2 cages, single-housed mice consumed approximately 40 g/kg/day of water (Fulenwider et al., 2024), whereas here they consumed an average of over 100 g/kg/day of water (Figure 3D). Consequently, preference for 6% ethanol was consistently around 70% in large unpartitioned cages, and slightly below 50% in the smaller, partitioned cages in the present study (Figure 3F). This comparison between studies suggests that decreasing the available area of HM2 cages, as we have done here, made the mice less discriminating in their choice of fluid. The reason for this effect is not clear and would need additional parametric experiments. For example, it would be useful to test whether this observation would apply to 2 BC procedures with other fluids, or even just with water alone. In any case, equalizing the space available to each mouse in HM2 cages allowed us to investigate the social facilitation of alcohol preference in the current experiments.

Specifically, we observed that while the intake of ethanol was on average nominally higher in socially housed versus single-housed mice in HM2 cages, this difference did not reach statistical significance (Figures 3A,B). In contrast, alcohol preference was significantly higher in the group-housed versus single-housed mice (Figures 3E,F). This difference in preference was primarily driven by a difference in water consumption, which was lower in socially housed versus single-housed mice (Figures 3C,D). These differences in preference and water intake were independent of sex or concentration of ethanol in the 2 BC procedure. The differences were also consistent across the circadian cycle, where hourly analysis of consumption showed nominally higher ethanol intake and lower water intake in socially housed mice during the dark phase of the circadian cycle (Figure 8). The relationship of fluid consumption reversed between housing conditions only at 1–2 h during the dark cycle, which could not have contributed to the overall differences in intake between groups.

While differences in ethanol intake between single and socially house mice in HM2 cages did not reach statistical significance, the number of entries into the ethanol channels when animals consumed alcohol (CVs) was significantly higher in group-housed versus single-housed mice during the 6% ethanol period (Figure 4B). Times spent in the channels (CV time) showed similar group differences as in analyses of CVs, but were complicated by sex differences, where socially housed males spent more time in the ethanol channels than females (Figure 5A). Overall, the group differences in CV times were less robust than differences in CVs (Figures 4, 5). Since CV times is a composite measure comprised of number of visits to the channel and the duration of each visit, these data indicate that differences in fluid consumption in this procedure are primarily driven by the number of visits to specific channels. Importantly, the increase in CVs into the ethanol channel was not accompanied by an increase in the number of visits when animals were not consuming ethanol (NNVs). In fact, NNVs to both ethanol and water channels were lower in socially housed versus single-housed mice (Figures 6A,B). The latter observation serves to indicate that the increase in ethanol CV is not a consequence of any potential locomotor stimulating effects of alcohol consumption or social housing.

Taken together, analyses of behavior in HM2 cages showing increased alcohol preference, decreased water intake, increased number of CVs to the ethanol channel, and decreased NNVs to both channels, suggest that socially housed mice were more deliberate in their attempts to access the ethanol channel, which can reflect higher motivation to access ethanol, than single-housed mice. It is important to consider, therefore, why socially housed mice could have higher motivation to access ethanol than single-housed mice. One possibility is that individual mice in social cages could exhibit individual influences on their cagemates to encourage their motivation for alcohol. We have observed such influences previously in other species of rodents. Specifically, we reported that pair-housed prairie voles *(Microtus ochrogaster)* can coordinate their alcohol intake resulting in significant correlations of alcohol intake between each member of a pair (Anacker et al., 2011a,b; Anacker and Ryabinin, 2013). This coordination of intake likely contributes to a high incidence of “cage effects” in prairie voles housed in HM2 cages at four per cage. In other words, prairie voles housed at four per cage are likely to contain either only high or only low alcohol-consuming animals (Walcott and Ryabinin, 2021). However, social behaviors are known to vastly differ between prairie voles and mice. Thus, while mice do prefer a social environment to isolation (Panksepp and Lahvis, 2007), in contrast to voles (and humans), they do not form long-term attachments between specific adult individuals (Insel et al., 1998; Young et al., 1999; Charles et al., 2014). In accordance, the social environment could influence alcohol drinking in these two species via different mechanisms. In agreement with this idea, comparing the distribution of high-, low- and medium-consuming individuals between cages with socially housed mice did not identify a strong clustering of mice with similar drinking levels (Figure 9). While there were differences between cages, the majority of cages contained animals with different levels of alcohol intake and preferences. Therefore, it seems likely that the increased motivation to consume ethanol in mice socially housed in HM2 cages could not be attributed to animals within each cage mimicking each other’s drinking. Instead, a more likely explanation is that the overall context of the presence or absence of cagemates influenced alcohol preference. At this point, it is difficult to speculate about precise neural mechanisms that could contribute to the effects of social context on alcohol drinking in HM2 cages. While there has been much research describing differences in gene expression and neurocircuits between socially and single-housed animals (Siuda et al., 2014; Yorgason et al., 2016; Zilkha and Kimchi, 2018), previous studies have been primarily performed in the context of standard housing and might not apply to conditions found in HM2 cages.

Although the presence of animals with varied alcohol intake within the same cage of socially housed mice suggests a lack of matching of drinking behavior between animals, it does not mean that individual animals in the cage do not influence each other’s drinking. Thus, differences in alcohol intake could be not due to mimicking behavior, but due to their different rank within a social hierarchy. Social hierarchies have been shown to influence alcohol intake in humans (Dumas et al., 2018), non-human primates (McKenzie-Quirk and Miczek, 2008), rats (Duncan et al., 2006), prairie voles (Anacker et al., 2014), and mice (Hilakivi-Clarke and Lister, 1992). However, rodent studies on the relationship between alcohol drinking and social hierarchy have not been performed in the context of HM2 cages. Our previous studies using the tube test of social dominance indicated that it usually takes more than 10 days to establish stable social hierarchies in mice socially housed in HM2 cages (Fulenwider et al., 2024). Confoundingly, the establishment of hierarchies in a social environment is promoted by tube testing (Zhou et al., 2017). Therefore, it is not clear whether the total of 10 days of housing in HM2 cages (5 days of drinking water and 5 days of 2 BC with ethanol) without repeated testing for social dominance, as was done here, was sufficient to result in a social hierarchy. Moreover, the social dominance ranking established by the tube test in HM2-housed mice in our previous study was not easily translatable to differences in consumption of sucrose, possibly due to ease of finding sufficient time to access this palatable fluid. Although alcohol and sucrose consumption could be differently regulated by social rank, it is unclear whether social hierarchies have contributed to differences in alcohol drinking within each cage of socially housed mice in our study.

Furthermore, being able to socially housed mice when testing individual fluid consumption is progress over testing alcohol intake in mice singly housed in shoebox cages, the HM2 cage analysis of drinking also has caveats. First, as mentioned previously, intakes in this procedure are not very high and the alcohol exposure only lasted 5 days. Therefore, interpreting these studies as addressing mechanisms of development of AUD can be questioned. In pilot experiments performed in our laboratory, we did not observe increases in alcohol intake when mice in HM2 cages were consuming alcohol for approximately 1 month or if the 2 BC procedure was preceded by “no choice” alcohol exposure (data not shown). These pilot experiments suggest that alcohol intakes and associated behaviors observed here are likely not to differ from the ones that could be achieved if additional paradigms thought to induce dependence in mice were employed. Therefore, the social effects of alcohol consumption studied here are more likely to model what is considered normative social drinking. Population statistics indicate that while 84% of the adult US population consumed alcoholic beverages at some point in their lifetime, only 6.3% reported current heavy alcohol use (SAMHSA, 2022a,b). We calculate that the top 6% of mice in our HM2 experiments consume an average of 9.5 g/kg/day. This is a substantial intake of ethanol, comparable to that observed in 2 BC procedures with higher concentrations of ethanol in mice individually housed in shoebox cages (McClearn and Rodgers, 1959; Belknap et al., 1993; Seemiller et al., 2022). Such doses consumed in 2 BC procedures are capable of producing hyperalgesia upon withdrawal following less than 2 weeks of exposure, which is a sign of at least mild alcohol dependence (Smith et al., 2016, 2017). Thus, future studies focusing on subpopulations of individual mice consuming high doses of alcohol in HM2 cages could be translationally important.

Another potential caveat of studies in HM2 cages is that at a time when mice are consuming fluid, they are in the channels by themselves, facing away from other mice in the cage. This situation is different from when human subjects are usually engaged in social drinking. It is not clear whether this difference plays a substantial role in regulating alcohol consumption. However, we note that prairie voles socially housed in HM2 cages consume very similar amounts of alcohol as when they are singly housed or pair-housed in shoebox cages (Anacker et al., 2011a; Walcott and Ryabinin, 2021). Therefore, at least in a species with translational validity for human social behaviors, this theoretical deficiency in the experimental setup of HM2 cages does not contribute to changes in alcohol drinking.

Despite the two caveats above, assessing ethanol intake in mice socially housed in HM2 cages provides substantial advantages over traditional preclinical methods used in studies on AUD. Efforts to allow testing alcohol drinking in socially housed animals via various other methods are underway in other research groups [reviewed in Ryabinin and Walcott (2018)]. While many of these methods have their own advantages, we note that currently they are based on either measuring proximity to the drinking spout (Logue et al., 2014; Varlinskaya et al., 2015) or registering licking behavior (Holgate et al., 2017; Stefaniuk et al., 2017; Frie and Khokhar, 2024). Although applying varied methods to assess behaviors has advantages over a single method, our studies in HM2 cages indicate that results from measures of approach to the fluid do not always coincide with measures of intake. Thus, although 6% ethanol CVs were significantly different between single and group-housed animals (Figure 4B), ethanol intakes were not significantly different between these groups (Figure 3B). In contrast, while water CVs at 3% ethanol were not significantly different between the groups (Figure 4C), water intake was significantly different between single and group-housed mice (Figure 3C). These findings reiterate the importance of distinguishing between the appetitive and consummatory phases of self-administration behaviors (Craig, 1917; Kringelbach and Berridge, 2016; Sherrington, 2023). Measuring just approach behavior or just intake could lead to different conclusions. Future studies should strive to incorporate analyses of both phases of alcohol consumption into their analysis.

Taken together, our experiments in standard housing conditions and automated HM2 cages, show that the effects of social housing on alcohol drinking depend on non-social parameters of the housing environment. We demonstrate that social housing increases alcohol preference and approach to the alcohol solution in environments requiring more deliberate effort to access consumed fluids. It will be important to start identifying molecular mechanisms underlying the potentially increased motivation to consume alcohol under these social circumstances. Identification of such mechanisms could help design therapeutic strategies to decrease problematic patterns of alcohol consumption in subjects with AUD.

## Data availability statement

The raw data supporting the conclusions of this article will be made available by the authors, without undue reservation.

## Ethics statement

The animal study was approved by Institutional Animal Care and Use Committee at OHSU, Portland, OR, United States. The study was conducted in accordance with the local legislation and institutional requirements.

## Author contributions

MJ: Data curation, Formal analysis, Investigation, Visualization, Writing – original draft. JZ: Data curation, Formal analysis, Investigation, Methodology, Writing – original draft. YZ: Investigation, Methodology, Writing – review & editing. AR: Conceptualization, Funding acquisition, Methodology, Project administration, Resources, Supervision, Writing – original draft.

## References

[ref1] Advani T. Hensler J. G. Koek W. (2007). Effect of early rearing conditions on alcohol drinking and 5-HT1A receptor function in C57BL/6J mice. Int. J. Neuropsychopharmacol. 10, 595–607. doi: 10.1017/S1461145706007401, PMID: 17134528

[ref2] Alcohol-Related Disease Impact. CDC [WWW Document]. (2023). Available at: https://nccd.cdc.gov/DPH_ARDI/Default/Default.aspx (Accessed 27 February, 2023).

[ref3] Anacker A. M. J. Loftis J. M. Kaur S. Ryabinin A. E. (2011a). Prairie voles as a novel model of socially-facilitated excessive drinking. Addict. Biol. 16, 92–107. doi: 10.1111/j.1369-1600.2010.00234.x, PMID: 20579002 PMC2950896

[ref4] Anacker A. M. J. Loftis J. M. Ryabinin A. E. (2011b). Alcohol intake in prairie voles is influenced by the drinking level of a peer. Alcohol. Clin. Exp. Res. 35, 1884–1890. doi: 10.1111/j.1530-0277.2011.01533.x, PMID: 21575019 PMC3158264

[ref5] Anacker A. Ryabinin A. (2013). Identification of subpopulations of prairie voles differentially susceptible to peer influence to decrease high alcohol intake. Front. Pharmacol. 4:84. doi: 10.3389/fphar.2013.00084 23847535 PMC3701123

[ref6] Anacker A. M. J. Smith M. L. Ryabinin A. E. (2014). Establishment of stable dominance interactions in prairie vole peers: relationships with alcohol drinking and activation of the paraventricular nucleus of the hypothalamus. Soc. Neurosci. 9, 484–494. doi: 10.1080/17470919.2014.931885, PMID: 24963825 PMC4349411

[ref7] Belknap J. K. Crabbe J. C. Young E. R. (1993). Voluntary consumption of ethanol in 15 inbred mouse strains. Psychopharmacology 112, 503–510. doi: 10.1007/BF02244901 7871064

[ref8] Berry A. Bellisario V. Capoccia S. Tirassa P. Calza A. Alleva E.. (2012). Social deprivation stress is a triggering factor for the emergence of anxiety- and depression-like behaviours and leads to reduced brain BDNF levels in C57BL/6J mice. Psychoneuroendocrinology 37, 762–772. doi: 10.1016/j.psyneuen.2011.09.007, PMID: 21974975

[ref9] Camarini R. Marianno P. Rae M. (2018). Social factors in ethanol sensitization. Int. Rev. Neurobiol. 140, 53–80. doi: 10.1016/bs.irn.2018.07.003, PMID: 30193709

[ref10] Caruso M. A. Robins M. T. Fulenwider H. D. Ryabinin A. E. (2021). Temporal analysis of individual ethanol consumption in socially housed mice and the effects of oxytocin. Psychopharmacology 238, 899–911. doi: 10.1007/s00213-020-05741-3, PMID: 33404737 PMC7786142

[ref11] Charles R. Sakurai T. Takahashi N. Elder G. A. Gama Sosa M. A. Young L. J.. (2014). Introduction of the human AVPR1A gene substantially alters brain receptor expression patterns and enhances aspects of social behavior in transgenic mice. Dis. Model. Mech. 7, 1013–1022. doi: 10.1242/dmm.017053, PMID: 24924430 PMC4107330

[ref12] Craig W. (1917). Appetites and aversions as constituents of instincts. Proc. Natl. Acad. Sci. USA 3, 685–688. doi: 10.1073/pnas.3.12.685, PMID: 16586767 PMC1091358

[ref13] Creswell K. G. (2021). Drinking together and drinking alone: a social-contextual framework for examining risk for alcohol use disorder. Curr. Dir. Psychol. Sci. 30, 19–25. doi: 10.1177/0963721420969406, PMID: 35291310 PMC8920309

[ref14] Dallas R. Field M. Jones A. Christiansen P. Rose A. Robinson E. (2014). Influenced but unaware: social influence on alcohol drinking among social acquaintances. Alcohol. Clin. Exp. Res. 38, 1448–1453. doi: 10.1111/acer.12375, PMID: 24588229

[ref15] Daly M. Robinson E. (2021). High-risk drinking in midlife before versus during the COVID-19 crisis: longitudinal evidence from the United Kingdom. Am. J. Prev. Med. 60, 294–297. doi: 10.1016/j.amepre.2020.09.004, PMID: 33234355 PMC7680033

[ref16] Deatherage G. (1972). Effects of housing density on alcohol intake in the rat. Physiol. Behav. 9, 55–57. doi: 10.1016/0031-9384(72)90264-8, PMID: 4673096

[ref17] Deutsch M. Gerard H. B. (1955). A study of normative and informational social influences upon individual judgement. J. Abnorm. Psychol. 51, 629–636. doi: 10.1037/h0046408 13286010

[ref18] Di Castelnuovo A. F. Costanzo S. de Gaetano G. (2019). Alcohol and the global burden of disease. Lancet 393:2389. doi: 10.1016/S0140-6736(19)30725-1 31204671

[ref19] Doty P. de Wit H. (1995). Effect of setting on the reinforcing and subjective effects of ethanol in social drinkers. Psychopharmacology 118, 19–27. doi: 10.1007/BF02245245, PMID: 7597118

[ref20] Dumas T. M. Davis J. P. Merrin G. J. Puccia M. Blustein D. (2018). If you’re high status and you know it: teasing apart the within- and between-person effects of peer- and self-reported status in the drinking group on alcohol-related outcomes. Psychol. Addict. Behav. J. Soc. Psychol. Addict. Behav. 32, 327–337. doi: 10.1037/adb0000352, PMID: 29578733

[ref21] Duncan E. A. Tamashiro K. L. K. Nguyen M. M. N. Gardner S. R. Woods S. C. Sakai R. R. (2006). The impact of moderate daily alcohol consumption on aggression and the formation of dominance hierarchies in rats. Psychopharmacology 189, 83–94. doi: 10.1007/s00213-006-0536-7, PMID: 16972102

[ref22] Ford M. M. Nickel J. D. Phillips T. J. Finn D. A. (2005). Neurosteroid modulators of GABA (a) receptors differentially modulate ethanol intake patterns in male C57BL/6J mice. Alcohol. Clin. Exp. Res. 29, 1630–1640. doi: 10.1097/01.alc.0000179413.82308.6b, PMID: 16205363 PMC1540354

[ref23] Frie J. A. Khokhar J. Y. (2024). FARESHARE: an open-source apparatus for assessing drinking microstructure in socially housed rats. NPP—Digital Psychiatry Neurosci. 2, 1–7. doi: 10.1038/s44277-024-00002-z PMC12624943 41361377

[ref24] Fulenwider H. D. Caruso M. A. Ryabinin A. E. (2022). Manifestations of domination: assessments of social dominance in rodents. Genes Brain Behav. 21:e12731. doi: 10.1111/gbb.12731, PMID: 33769667 PMC8464621

[ref25] Fulenwider H. D. Robins M. T. Caruso M. A. Ryabinin A. E. (2021). Social housing leads to increased ethanol intake in male mice housed in environmentally enriched cages. Front. Behav. Neurosci. 15:409. doi: 10.3389/fnbeh.2021.695409 PMC8253159 34220465

[ref26] Fulenwider H. D. Zhang Y. Ryabinin A. E. (2024). Characterization of social hierarchy formation and maintenance in same-sex, group-housed male and female C57BL/6 J mice. Horm. Behav. 157:105452. doi: 10.1016/j.yhbeh.2023.105452, PMID: 37977023 PMC10841988

[ref27] GBD (2016). Alcohol collaborators, 2018. Alcohol use and burden for 195 countries and territories, 1990-2016: a systematic analysis for the global burden of disease study 2016. Lancet Lond. Engl. 392, 1015–1035. doi: 10.1016/S0140-6736(18)31310-2, PMID: 30146330 PMC6148333

[ref28] Grant B. F. Chou S. P. Saha T. D. Pickering R. P. Kerridge B. T. Ruan W. J.. (2017). Prevalence of 12-month alcohol use, high-risk drinking, and DSM-IV alcohol use disorder in the United States, 2001-2002 to 2012-2013: results from the National Epidemiologic Survey on alcohol and related conditions. JAMA Psychiatry 74, 911–923. doi: 10.1001/jamapsychiatry.2017.2161, PMID: 28793133 PMC5710229

[ref29] Griffin W. C. Middaugh L. D. Becker H. C. (2007). Voluntary ethanol drinking in mice and ethanol concentrations in the nucleus accumbens. Brain Res. 1138, 208–213. doi: 10.1016/j.brainres.2006.12.071, PMID: 17275791

[ref30] Hashikawa K. Hashikawa Y. Lischinsky J. Lin D. (2018). The neural mechanisms of sexually dimorphic aggressive behaviors. Trends Genet. TIG 34, 755–776. doi: 10.1016/j.tig.2018.07.001, PMID: 30173869

[ref31] Heilig M. Epstein D. H. Nader M. A. Shaham Y. (2016). Time to connect: bringing social context into addiction neuroscience. Nat. Rev. Neurosci. 17, 592–599. doi: 10.1038/nrn.2016.67, PMID: 27277868 PMC5523661

[ref32] Hilakivi-Clarke L. Lister R. G. (1992). Social status and voluntary alcohol consumption in mice: interaction with stress. Psychopharmacology 108, 276–282. doi: 10.1007/BF02245112, PMID: 1523279

[ref33] Holgate J. Y. Garcia H. Chatterjee S. Bartlett S. E. (2017). Social and environmental enrichment has different effects on ethanol and sucrose consumption in mice. Brain Behav. 7:e00767. doi: 10.1002/brb3.767, PMID: 28828224 PMC5561324

[ref34] Ieraci A. Mallei A. Popoli M. (2016). Social isolation stress induces anxious-depressive-like behavior and alterations of neuroplasticity-related genes in adult male mice. Neural Plast. 2016, 6212983–6212913. doi: 10.1155/2016/6212983, PMID: 26881124 PMC4736811

[ref35] Insel T. R. Winslow J. T. Wang Z. Young L. J. (1998). Oxytocin, vasopressin, and the neuroendocrine basis of pair bond formation. Adv. Exp. Med. Biol. 449, 215–224. doi: 10.1007/978-1-4615-4871-3_28, PMID: 10026808

[ref36] Irizar P. Jones A. Christiansen P. Goodwin L. Gage S. H. Roberts C.. (2021). Longitudinal associations with alcohol consumption during the first COVID-19 lockdown: associations with mood, drinking motives, context of drinking, and mental health. Drug Alcohol Depend. 226:108913. doi: 10.1016/j.drugalcdep.2021.108913, PMID: 34315105 PMC8567536

[ref37] Karamihalev S. Brivio E. Flachskamm C. Stoffel R. Schmidt M. V. Chen A. (2020). Social dominance mediates behavioral adaptation to chronic stress in a sex-specific manner. eLife 9:e58723. doi: 10.7554/eLife.58723, PMID: 33034286 PMC7679136

[ref38] Killgore W. D. S. Cloonan S. A. Taylor E. C. Lucas D. A. Dailey N. S. (2021). Alcohol dependence during COVID-19 lockdowns. Psychiatry Res. 296:113676. doi: 10.1016/j.psychres.2020.113676, PMID: 33385782 PMC9754813

[ref39] Koskela M. Piepponen T. P. Andressoo J.-O. Võikar V. Airavaara M. (2018). Towards developing a model to study alcohol drinking and craving in female mice housed in automated cages. Behav. Brain Res 352, 116–124. doi: 10.1016/j.bbr.2018.03.027 29572104

[ref40] Kringelbach M. L. Berridge K. C. (2016). “Neuroscience of reward, motivation, and drive” in Recent developments in neuroscience research on human motivation, advances in motivation and achievement (Leeds, West Yorkshire, England: Emerald Group Publishing Limited).

[ref41] Krügel U. Fischer J. Bauer K. Sack U. Himmerich H. (2014). The impact of social isolation on immunological parameters in rats. Arch. Toxicol. 88, 853–855. doi: 10.1007/s00204-014-1203-0, PMID: 24500571

[ref42] Kuntsche E. Rehm J. Gmel G. (2004). Characteristics of binge drinkers in Europe. Soc. Sci. Med. 59, 113–127. doi: 10.1016/j.socscimed.2003.10.009 15087148

[ref43] Larsen H. Overbeek G. Granic I. Engels R. C. M. E. (2010). Imitation of alcohol consumption in same-sex and other-sex dyads. Alcohol Alcohol. Oxf. Oxfs. 45, 557–562. doi: 10.1093/alcalc/agq053, PMID: 20847061

[ref44] Logue S. Chein J. Gould T. Holliday E. Steinberg L. (2014). Adolescent mice, unlike adults, consume more alcohol in the presence of peers than alone. Dev. Sci. 17, 79–85. doi: 10.1111/desc.12101, PMID: 24341974 PMC3869041

[ref45] Lopez M. F. Doremus-Fitzwater T. L. Becker H. C. (2011). Chronic social isolation and chronic variable stress during early development induce later elevated ethanol intake in adult C57BL/6J mice. Alcohol Fayettev. N 45, 355–364. doi: 10.1016/j.alcohol.2010.08.017, PMID: 20880662 PMC3013234

[ref46] Lopez M. F. Laber K. (2015). Impact of social isolation and enriched environment during adolescence on voluntary ethanol intake and anxiety in C57BL/6J mice. Physiol. Behav. 148, 151–156. doi: 10.1016/j.physbeh.2014.11.012, PMID: 25446196 PMC4425642

[ref47] Mac Killop J. Agabio R. Feldstein-Ewing S. Heilig M. Kelly J. F. Leggio L.. (2022). Hazardous drinking and alcohol use disorders. Nat. Rev. Dis. Primer 8:80. doi: 10.1038/s41572-022-00406-1, PMID: 36550121 PMC10284465

[ref48] McClearn G. E. Rodgers D. A. (1959). Differences in alcohol preference among inbred strains of mice. Q. J. Stud. Alcohol 20, 691–695. doi: 10.15288/qjsa.1959.20.691

[ref49] McKenzie-Quirk S. D. Miczek K. A. (2008). Social rank and social separation as determinants of alcohol drinking in squirrel monkeys. Psychopharmacology 201, 137–145. doi: 10.1007/s00213-008-1256-y, PMID: 18641974 PMC4371730

[ref50] Monk R. L. Qureshi A. Heim D. (2020). An examination of the extent to which mood and context are associated with real-time alcohol consumption. Drug Alcohol Depend. 208:107880. doi: 10.1016/j.drugalcdep.2020.107880, PMID: 32004997

[ref51] Monk R. L. Qureshi A. W. McNeill A. Erskine-Shaw M. Heim D. (2018). Perfect for a gin and tonic: how context drives consumption within a modified bogus taste test. Alcohol Alcohol. Oxf. Oxfs. 53, 228–234. doi: 10.1093/alcalc/agx084, PMID: 29136090

[ref52] NIAAA (2024) Surveillance Report #120 | National Institute on Alcohol Abuse and Alcoholism (NIAAA) [WWW Document]. Available at: https://www.niaaa.nih.gov/publications/surveillance-reports/surveillance120 (Accessed 29 January, 2024).

[ref53] Nogueira-Arjona R. Shannon T. Kehayes I.-L. Sherry S. B. Keough M. T. Stewart S. H. (2019). Drinking to keep pace: a study of the moderating influence of extraversion on alcohol consumption similarity in drinking buddy dyads. Addict. Behav. 92, 69–75. doi: 10.1016/j.addbeh.2018.12.023, PMID: 30597333

[ref54] Norman K. J. Seiden J. A. Klickstein J. A. Han X. Hwa L. S. DeBold J. F.. (2015). Social stress and escalated drug self-administration in mice I. Alcohol and corticosterone. Psychopharmacology (Berl.) 232, 991–1001. doi: 10.1007/s00213-014-3733-9, PMID: 25242256 PMC4339510

[ref55] Panksepp J. B. Lahvis G. P. (2007). Social reward among juvenile mice. Genes Brain Behav. 6, 661–671. doi: 10.1111/j.1601-183X.2006.00295.x, PMID: 17212648 PMC2040181

[ref56] Panksepp J. B. Rodriguez E. D. Ryabinin A. E. (2017). Sweetened ethanol drinking during social isolation: enhanced intake, resistance to genetic heterogeneity and the emergence of a distinctive drinking pattern in adolescent mice. Genes Brain Behav. 16, 369–383. doi: 10.1111/gbb.12346, PMID: 27706910 PMC5334449

[ref57] Parker L. F. Radow B. L. (1974). Isolation stress and volitional ethanol consumption in the rat. Physiol. Behav. 12, 1–3. doi: 10.1016/0031-9384(74)90060-2, PMID: 4855694

[ref58] Pohorecky L. A. (2010). Acute novel stressors modify ethanol intake of psychosocially stressed rats. Pharmacol. Biochem. Behav. 95, 390–400. doi: 10.1016/j.pbb.2010.02.017, PMID: 20211641

[ref59] Quigley B. M. Collins R. L. (1999). The modeling of alcohol consumption: a meta-analytic review. J. Stud. Alcohol 60, 90–98. doi: 10.15288/jsa.1999.60.90 10096313

[ref60] Ryabinin A. E. Walcott A. T. (2018). Assessing social alcohol drinking in rodent models: are we there yet? Int. Rev. Neurobiol. 140, 33–51. doi: 10.1016/bs.irn.2018.07.002, PMID: 30193708

[ref61] Sacks J. J. Gonzales K. R. Bouchery E. E. Tomedi L. E. Brewer R. D. (2015). 2010 national and state costs of excessive alcohol consumption. Am. J. Prev. Med. 49, e73–e79. doi: 10.1016/j.amepre.2015.05.031, PMID: 26477807

[ref62] SAMHSA (2022a). Section 2 PE Tables – Results from the 2021 National Survey on Drug Use and Health: Detailed Tables, SAMHSA, CBHSQ [WWW Document]. Available at: https://www.samhsa.gov/data/sites/default/files/reports/rpt39441/NSDUHDetailedTabs2021/NSDUHDetailedTabs2021/NSDUHDetTabsSect2pe2021.htm#tab2.29b (Accessed 15 April, 2024).

[ref63] SAMHSA (2022b). Section 2 PE Tables – Results from the 2022 National Survey on Drug Use and Health: Detailed Tables, SAMHSA, CBHSQ [WWW Document]. Available at: https://www.samhsa.gov/data/sites/default/files/reports/rpt42728/NSDUHDetailedTabs2022/NSDUHDetailedTabs2022/NSDUHDetTabsSect2pe2022.htm#tab2.25a (Accessed 15 April, 2024).

[ref64] Seemiller L. R. Logue S. F. Gould T. J. (2022). Inbred mouse strain differences in alcohol and nicotine addiction-related phenotypes from adolescence to adulthood. Pharmacol. Biochem. Behav. 218:173429. doi: 10.1016/j.pbb.2022.173429, PMID: 35820468 PMC11524176

[ref65] Sherrington C. S. (2023). “The integrative action of the nervous system” in Scientific and medical knowledge production (New Haven: Yale University Press).

[ref66] Siuda D. Wu Z. Chen Y. Guo L. Linke M. Zechner U.. (2014). Social isolation-induced epigenetic changes in midbrain of adult mice. J. Physiol. Pharmacol. Off. J. Pol. Physiol. Soc. 65, 247–255. 24781734

[ref67] Smith M. L. Hostetler C. M. Heinricher M. M. Ryabinin A. E. (2016). Social transfer of pain in mice. Sci. Adv. 2:e1600855. doi: 10.1126/sciadv.1600855, PMID: 27774512 PMC5072181

[ref68] Smith M. L. Walcott A. T. Heinricher M. M. Ryabinin A. E. (2017). Anterior cingulate cortex contributes to alcohol withdrawal- induced and socially transferred hyperalgesia. eNeuro 4:17. doi: 10.1523/ENEURO.0087-17.2017, PMID: 28785727 PMC5526654

[ref69] Stefaniuk M. Beroun A. Lebitko T. Markina O. Leski S. Meyza K.. (2017). Matrix Metalloproteinase-9 and synaptic plasticity in the central amygdala in control of alcohol-seeking behavior. Biol. Psychiatry 81, 907–917. doi: 10.1016/j.biopsych.2016.12.026, PMID: 28190519

[ref70] Stefaniuk M. Pawłowska M. Barański M. Nowicka K. Zieliński Z. Bijoch Ł.. (2023). Global brain c-Fos profiling reveals major functional brain networks rearrangements after alcohol reexposure. Neurobiol. Dis. 178:106006. doi: 10.1016/j.nbd.2023.106006, PMID: 36682503

[ref71] Sudhinaraset M. Wigglesworth C. Takeuchi D. T. (2016). Social and cultural contexts of alcohol use. Alcohol Res. Curr. Rev. 38, 35–45. 10.35946/arcr.v38.1.05 PMC4872611 27159810

[ref72] Thiessen D. D. Rodgers D. A. (1965). Alcohol injection, grouping, and voluntary alcohol consumption of inbred strains of mice. Q. J. Stud. Alcohol 26, 378–383. doi: 10.15288/qjsa.1965.26.378, PMID: 5892891

[ref73] Thomsen M. Dencker D. Wörtwein G. Weikop P. Egecioglu E. Jerlhag E.. (2017). The glucagon-like peptide 1 receptor agonist Exendin-4 decreases relapse-like drinking in socially housed mice. Pharmacol. Biochem. Behav. 160, 14–20. doi: 10.1016/j.pbb.2017.07.014, PMID: 28778739

[ref74] Tomaszewski R. J. Strickler D. P. Maxwell W. A. (1980). Influence of social setting and social drinking stimuli on drinking behavior. Addict. Behav. 5, 235–240. doi: 10.1016/0306-4603(80)90045-3, PMID: 7435311

[ref75] van Erp A. M. Tachi N. Miczek K. A. (2001). Short or continuous social stress: suppression of continuously available ethanol intake in subordinate rats. Behav. Pharmacol. 12, 335–342. doi: 10.1097/00008877-200109000-00004 11710748

[ref76] Varlinskaya E. I. Truxell E. M. Spear L. P. (2015). Ethanol intake under social circumstances or alone in Sprague-dawley rats: impact of age, sex, social activity, and social anxiety-like behavior. Alcohol. Clin. Exp. Res. 39, 117–125. doi: 10.1111/acer.12604, PMID: 25623411 PMC4308818

[ref77] Venniro M. Banks M. L. Heilig M. Epstein D. H. Shaham Y. (2020). Improving translation of animal models of addiction and relapse by reverse translation. Nat. Rev. Neurosci. 21, 625–643. doi: 10.1038/s41583-020-0378-z, PMID: 33024318

[ref78] Walcott A. T. Ryabinin A. E. (2021). Assessing effects of oxytocin on alcohol consumption in socially housed prairie voles using radio frequency tracking. Addict. Biol. 26:e12893. doi: 10.1111/adb.12893, PMID: 32160654 PMC7483374

[ref79] Weerakoon S. M. Jetelina K. K. Knell G. (2021). Longer time spent at home during COVID-19 pandemic is associated with binge drinking among US adults. Am. J. Drug Alcohol Abuse 47, 98–106. doi: 10.1080/00952990.2020.1832508, PMID: 33280423

[ref80] Weitzman E. R. Nelson T. F. Wechsler H. (2003). Taking up binge drinking in college: the influences of person, social group, and environment. J. Adolesc. Health 32, 26–35. doi: 10.1016/S1054-139X(02)00457-3, PMID: 12507798

[ref81] Yorgason J. T. Calipari E. S. Ferris M. J. Karkhanis A. N. Fordahl S. C. Weiner J. L.. (2016). Social isolation rearing increases dopamine uptake and psychostimulant potency in the striatum. Neuropharmacology 101, 471–479. doi: 10.1016/j.neuropharm.2015.10.025, PMID: 26525189 PMC4681685

[ref82] Young L. J. Nilsen R. Waymire K. G. Mac Gregor G. R. Insel T. R. (1999). Increased affiliative response to vasopressin in mice expressing the V1a receptor from a monogamous vole. Nature 400, 766–768. doi: 10.1038/23475, PMID: 10466725

[ref83] Zhou T. Zhu H. Fan Z. Wang F. Chen Y. Liang H.. (2017). History of winning remodels thalamo-PFC circuit to reinforce social dominance. Science 357, 162–168. doi: 10.1126/science.aak9726, PMID: 28706064

[ref84] Zilkha N. Kimchi T. (2018). A molecular signature for social isolation identified in the brain. Nature 559, 38–40. doi: 10.1038/d41586-018-05447-9, PMID: 29959409

